# Scales Tell a Story on the Stress History of Fish

**DOI:** 10.1371/journal.pone.0123411

**Published:** 2015-04-29

**Authors:** Johan Aerts, Juriaan Rogier Metz, Bart Ampe, Annemie Decostere, Gert Flik, Sarah De Saeger

**Affiliations:** 1 Bio-analysis Research Group, Animal Sciences Unit, Institute for Agriculture and Fisheries Research, Melle, Belgium; 2 Laboratory of Food Analysis, Department of Bio-analysis, Faculty of Pharmaceutical Sciences, Ghent University, Ghent, Belgium; 3 Department of Animal Physiology, Institute for Water and Wetland Research, Faculty of Science, Radboud University Nijmegen, Nijmegen, The Netherlands; 4 Biostatistics and Data modeling, Animal Sciences Unit, Institute for Agriculture and Fisheries Research, Melle, Belgium; 5 Department of Morphology, Faculty of Veterinary Medicine, Ghent University, Ghent, Belgium; Sonoma State University, UNITED STATES

## Abstract

Fish faced with stressful stimuli launch an endocrine stress response through activation of the hypothalamic-pituitary-interrenal (HPI-) axis to release cortisol into the blood. Scientifically validated biomarkers to capture systemic cortisol exposure over longer periods of time are of utmost importance to assess chronic stress in governmental, wildlife, aquaculture and scientific settings. Here we demonstrate that cortisol in scales of common carp (*Cyprinus carpio* L.) is the long-sought biomarker for chronic stress. Undisturbed (CTR) and daily stressed (STRESS) carp were compared. Dexamethasone (DEX) or cortisol (CORT) fed fish served as negative and positive controls, respectively. Scale cortisol was quantified with a validated ultra-performance liquid chromatography tandem mass spectrometry method. An increase in scale cortisol content was found in STRESS and CORT but not in CTR and DEX fish. Scale cortisol content reflects its accumulation in a stressor and time dependent manner and validates the scale cortisol content as biomarker for chronic stress. Plasma analyses confirmed that (i) CTR, DEX and CORT treatments were effective, (ii) plasma cortisol of STRESS fish showed no signs of chronic HPI-axis activation, and (iii) plasma cortisol is a poor predictor for chronic stress. The expression of HPI key genes *crf*, *pomc*, and *star* were up-regulated in STRESS fish in the absence of a plasma cortisol response, as was the target gene of cortisol encoding subunit α1 of the Na^+^/K^+^-ATPase in gills. When lost, scales of fish regenerate fast. Regenerated scales corroborate our findings, offering (i) unsurpassed time resolution for cortisol incorporation and as such for stressful events, and (ii) the possibility to investigate stress in a well defined and controlled environment and time frame creating novel opportunities for bone physiological research. We conclude that the cortisol content in ontogenetic and regenerated scales is an innovative biomarker for chronic stress offering ample applications in science and industry.

## Introduction

Fish increasingly attract public, scientific and political interest around the world. Recreational and commercial fisheries are prominent in our societies; fish issues take a highly relevant position in discussions related to conservation biology [1] and environmental protection efforts (*e*.*g*. effects of climate change, novel predators, novel animal-environment relationships on stress and as a consequence on fitness) [2–3]. Anthropogenic activities (*e*.*g*. energy production, shipping traffic, industrial pollution) compromise wild stocks [4]. Therefore, various international monitoring schemes aim to scientifically clarify their impact on the health status of oceanic niches. A similar situation exists for freshwater stocks as they are under threat by soil erosion, fertilizers, etc. as agriculture expands. The human population keeps on expanding, making the need for a sustainable food production a global prime priority. Fish protein is one of the most important protein sources for human consumption. Now that fisheries meet limits in yield, aquaculture expands rapidly worldwide and puts increasing pressure on farmers to produce in an optimal, sustainable and animal friendly way [5–6]. High numbers of fish (*e*.*g*. zebrafish and medaka) are used as vertebrate models and alternatives to rodents in biomedical research (*e*.*g*. in bone physiological research). The imperative to maintain research-driven innovation in this sector, while global economic output dwindles, adds to exploitation of fish. In the recent past ethics concerning fish welfare urge more attention as high numbers of fish are involved in fisheries [7], the rapidly growing and intensifying aquaculture industries [8–9], public aquaria [10–11] and scientific research laboratories [12]. Appreciating and understanding fish biology as basis for management, control and decision-making in fish exploitation puts a phenomenal challenge to those involved, coming from a multitude of disciplines (from molecular biology to eco-physiology), viewing from multiple angles (all different stakeholders) and representing highly specific expertise. In this framework new scientifically validated biomarkers to assess levels of stress in fish, in particular of chronic stress, are of utmost importance.

Fish welfare easily becomes compromised, and consensus is growing that proper welfare assessment requires animal-based, physiological indicators being superior to less consistent, indirect husbandry-related parameters such as water quality. However, a shortage exists on practical, reliable and validated biomarkers for chronic stress. A frequently used, seemingly logical biomarker for fish is the blood level of the ‘stress steroid’ cortisol. Fish faced with stressful stimuli launch an endocrine stress response through activation of the hypothalamic-pituitary-interrenal (HPI-) axis to release cortisol [13–14] into the blood. Cortisol elicits a suite of physiological and behavioral changes [15–17] that allow the fish to cope with altered situations [18–20]. The adaptive value of short-term cortisol actions is widely recognized [21–22]. Far less is known about persistent stress and its mostly detrimental consequences for health, growth, and reproduction [19–20]. Definition of a robust, easily performable and scientifically validated chronic stress biomarker that captures systemic cortisol exposure over longer periods of time is thus of utmost importance. Glucocorticoid levels in plasma of fish often show diel variation [23], do not reveal the lifetime exposure of the fish to stress, and provide no more than a snapshot of the cortisol status at the moment of sampling [24–25]. Moreover, blood sampling is invasive and unavoidably causes confounding stress to the fish because of netting, air exposure and handling. This makes plasma cortisol prone to bias as levels rise rapidly a few minutes after confrontation with a stressor [13]. Anesthetics adopted to facilitate blood sampling may by themselves reduce or block the activation of the HPI-axis, thereby affecting the cortisol release into the blood and resulting in erroneous results [26–27]. Restrictions also apply to the assay of cortisol in alternative matrices such as mucus [28–29], gut content [29], feces [30] and water [31–32]. The pertinent literature lacks data on cortisol in a matrix that captures systemic cortisol exposure over longer periods of time suitable for chronic stress evaluation. Hitherto, the majority of studies addressed cortisol only, and reports on glucocorticoid production pathway(s) [33] or their significance in chronic stress are scarce. Fish record (a-)biotic events and store this history in calcified tissues [34–37]. As is the case for feathers of birds [38] and hair of mammals [39], the ideal matrix for chronic stress assessment in fish should at least meet the following criteria: (i) slow but persistent growth, (ii) incorporation of glucocorticoids, and (iii) ease in sampling.

Here we show, based on our findings in common carp (*Cyprinus carpio* L.), that elasmoid scales of teleostean fish meet the criteria of an ideal matrix for chronic stress assessment. Elasmoid scales, calcified dermal exoskeletal structures [40], grow along with the fish and consist of an acellular collagenous matrix, which is mineralized with calcium hydroxyapatite on the outer layer and lined with a monolayer of cells with osteoblast- and osteoclast-like properties. Upon removal, a scale will regenerate within days [41]. Scales are a target for endocrine stimuli. A high affinity, low-capacity estradiol-17b binding was found in scleroblast cytosol of rainbow trout (*Oncorhynchus mykiss*) [42] and estrogen receptors have immunohistochemically been detected in Mozambique tilapia (*Oreochromis mossambicus*) and gilthead sea bream (*Sparus auratus*) scales [43]. A scale is easily and quickly collected with negligible injury and stress to the fish without confounding its cortisol levels due to the sampling procedure. In line with our initial hypothesis, the scale cortisol content proved to reflect the stress history of the fish [44]. This long-sought biomarker captures systemic cortisol exposure over longer periods of time making it suitable to quantify chronic stress in fish. It will help monitoring of general health of wild stocks, developing sustainable aquaculture, secure fish performance in public aquaria and scientific research, and will allow more in-depth physiological research of calcified structures.

## Materials and Methods

### Experimental set-up

Experimental procedures were according to Dutch legislation and approved by the Animal Ethics Committee of the Radboud University (permit number: RU-DEC2013-192). Common carp were obtained from a commercial farm (Viskweekcentrum Valkenswaard, The Netherlands). Fish were reared to adulthood at the Radboud University of Nijmegen to ensure the history in glass tanks containing 140 L recirculated (80%; 20% fresh input of tap water), biofiltered and UV-treated Nijmegen tap water at 20°C. Growing adult fish (at day 21: 340 ± 92 g and 24.6 ± 2.4 cm; at day 42: 377 ± 108 g and 25.4 ± 2.5 cm), held under 12:12 h light:dark, fed commercial fish feed pellets (Skretting, Stavanger, Norway) 2 meals a day (09.30 am and 03.30 pm) at 0.9% of body weight were kept at 20.0 ± 0.4°C for 6 weeks. Four groups, with 2 tank replicates per group, of 6 fish per tank each were set up. Fish of the CTR group were left undisturbed. The DEX and CORT groups received feed spiked with 500 mg kg^-1^ dexamethasone (Sigma-Aldrich, St. Louis, MO, USA) and cortisol (hydrocortisone, Sigma-Aldrich, St. Louis, MO, USA), respectively, for the entire 6-weeks duration of the experiment. Spiked feeds were prepared by uniformly spraying pellets with DEX or CORT (1 mg ml^-1^ in ethanol); pellets were left to dry overnight at room temperature [45]. The STRESS group was stressed once daily. The type, duration and timing of a subset of stressors were applied randomly and included: netting (15–60 min), air exposure (1–3 min), sudden temperature drop (up to 5°C), chasing (up to 10 min) and confinement (in a bucket with low water level).

Twenty-one days after the start of the experiment, fish were anesthesized in 2-phenoxy-ethanol (0.1%; v/v) and 10 scales were collected from the left flank of the fish. Fish were allowed to recover and placed back in the respective tanks. After 42 days fish were anesthesized 45 min after feeding and blood was collected by puncture of the caudal vein with a tuberculin syringe fitted with a 25-G needle; heparin (LEO Pharma, Ballerup, Danmark) was used as anticoagulant. After blood collection, the fish were sacrificed by cutting the spinal cord caudal to the opercula. Ontogenetic scales (from the right flank) as well as the 21-days regenerated scales (from the left flank) were collected.

Fulton’s condition factor (K factor; 100*W/L^3^, where W is whole body wet weight in grams and L is standard length in centimeters) was calculated for each fish [46].

### Plasma analyses

Plasma cortisol [47] was measured in duplicate using a radio immunoassay (RIA) in a 96-well plate. The primary antibody (Cortisol Antibody [xm210] monoclonal and IgG purified) (Abcam, Cambridge, United Kingdom) shows a 100% cross reactivity with cortisol, 0.9% with 11-deoxycortisol, 0.6% with corticosterone, and < 0.01% with 11-deoxycorticosterone, progesterone, 17α-hydroxyprogesterone, testosterone and estradiol. Since this RIA is not suitable for cortisol determination in plasma of DEX fish, as the antibody cross reacts with DEX, plasma of DEX fish was measured using a commercial cortisol enzyme-linked immunosorbent assay (ELISA) kit (Abcam #ab108665). All wells except the ‘non-specifics’ were coated with 100 μl cortisol (1:2,000 in 50 mM NaHCO_3_, 50 mM NaH_2_CO_3_, 0.02% NaN_3_, pH = 9.6) and incubated overnight at 4°C. The following day, the plates were washed three times with 200 μl/well (wash buffer: 100 mM Tris, 0.9% NaCl, 0.02% NaN_3_, pH = 7.4). Subsequently, non-specific sites were blocked by the addition of 100 μl blocking buffer (wash buffer supplemented with 0.25% (v/v) Normal Calf Serum) to each well. Plates were covered and incubated for 1 h at 37°C. Subsequently, 10 μl of standard (4 pg—2048 pg cortisol/10 μl assay buffer containing 100 mM Tris, 0.9% NaCl, 0.1% 8-anilino-1-naphthalenesulfonic acid, 0.02% NaN_3_) or 10 μl of undiluted plasma was added to designated wells. Non-specifics and B0 received 10 μl assay buffer. After the addition of standards and samples, 90 μl ^3^H-hydrocortisone (PerkinElmer, USA, 1:10,000 in assay buffer) solution was added to all wells. Plates were incubated overnight at 4°C. The plates were then washed three times with wash buffer. After the final wash step, all wells received 200 μl scintillation liquid (Optiphase hisafe-3, PerkinElmer, USA) and were covered. Beta-emission was quantified by a 3 min count per well using a Microbeta Plus (Wallac/PerkinElmer, USA). Inter- and intra-assay variations were 12.5% and 3.5%, respectively.

Enzymatic colorimetric determination of glucose was done according to the GOD-PAP method [48]. Ten μl water (blank), standard or undiluted plasma (all in triplicate) was pipetted in a 96-well plate and 200 μl color reagent was added to each well. Absorbance at 490 nm was measured and read against a standard curve (three dilutions of a glucose standard were used: 1.38 mM, 2.78 mM, and 5.55 mM).

Plasma lactate was measured with a commercially available test kit (Instruchemie, Delfzijl, The Netherlands), with the protocol adapted to 96-well microtiter plates [48]. Ten μl sample, standard or blank (all in duplicate) was mixed with 290 μl lactate reagent and incubated for 20 min at 37°C. Absorbance at 455 nm was measured and read against a standard curve.

Plasma total calcium was measured using the *o*-cresolphthalein method [49]. Ten μl of 1:10 diluted plasma, standard or blank was used (all in triplicate) and mixed with 200 μl color reagent. Color reagent was prepared freshly by mixing 80% water with 10% calcium buffer (3.5 M 2-amino-2-methylpropan-1-ol, pH = 10.7) and 10% chromogen solution (0.15 mM phthalein purple, 6.9 M 8-hydroxyquinoline, 60 mM HCl). After 5 min incubation at room temperature, absorbance at 570 nm was measured spectrophotometrically.

Plasma osmolality was determined using a cryoscopic osmometer (Osmomat 030, Gonotec, Berlin, Germany) [48].

### Scale cortisol analyses

An ultra-performance liquid chromatography tandem mass spectrometry (UPLC-MS/MS) quantification method for cortisol in fish scales was developed in an EN ISO/IEC 17025 [50] accredited environment and validated according the requirements of the Commission Decision No. 2002/657/EC [51].

Chromatographic analysis was performed on an Acquity UPLC-MS/MS Premier XE using an Acquity Ultra Performance LC BEH C_18_ (1.7 μm; 2.1 mm x 100 mm) column (Waters, Milford, USA). Samples were evaporated to dryness with a Turbovap nitrogen evaporator (Biotage, Sweden). Grace Pure SPE C18-Max (500 mg, 6 ml) columns for solid-phase extraction (SPE) were obtained from Grace Davison Discovery Sciences (Lokeren, Belgium). High-performance liquid chromatography (HPLC)-gradient grade methanol (Hipersolv Chromanorm) as extraction solvent was obtained from VWR International BVBA (Leuven, Belgium). Methanol absolute LC-MS as well as formic acid ULC-MS grade from Biosolve BV (Valkenswaard, The Netherlands) and ultrapure water of a Milli-Q gradient Q-Gard 2 from Millipore (Billerica, USA) were used as mobile phase solvents.

Only products with a certificate of analysis were used. Cortisol was from Sigma-Aldrich (Diegem, Belgium) and cortisol-d_4_ from CDN Isotopes (Pointe-Claire, Canada) was used as an internal standard. Individual stock standard solutions of 1 mg mL^-1^ of cortisol and internal standard were prepared in methanol and stored at 4°C. Calibration standards, ranging from 5 μg L^-1^ to 100 μg L^-1^, were prepared by addition of 10 μl of a cortisol-d_4_ solution of 0.5 μg L^-1^ to 0.5 μL, 1 μL, 2.5 μL, 5 μL, and 10 μL of a cortisol standard solution of 1 μg L^-1^ in 100 μL of H_2_O/MeOH (80:20; v/v), respectively. As 100 mg of sample were used, this corresponded to a range from 5 μg kg^-1^ to 100 μg kg^-1^ in fish scales.

Scales were sampled from sacrificed fish deeply anesthesized with 2-phenoxy-ethanol (0.1%; v/v) (Sigma-Aldrich, St. Louis, USA). Scales from a standardized row dorsally to the lateral line were removed from the skin with fine tweezers. All samples were rinsed with ultrapure water and air dried on a paper tissue at room temperature.

To obtain a homogenized sample scales were cut into fine pieces using scissors. Between samples, scissors were rinsed with ethanol followed by ultrapure water and dried with a paper tissue to avoid cross-contamination between samples. Of the homogenized sample 0.100 ± 0.001 g was weighed into a 10 ml test tube. Subsequently, 8 ml of methanol was added as extraction solvent and 10 μL of a cortisol-d_4_ solution of 0.5 μg L^-1^ was added as internal standard. When lower amounts of scale were used, the volume of cortisol-d_4_ was adapted accordingly. The sample was vortex-mixed for 30 s, placed on an overhead shaker at 60 rpm for 1 h at room temperature, and centrifuged for 10 min at 3500 g at 7°C. All supernatant was taken, evaporated to dryness under nitrogen at 60°C using a nitrogen evaporator, and reconstituted in 5 ml H_2_O/MeOH (80:20; v/v). After conditioning a C_18_ SPE column with 3 ml of methanol followed by 3 ml of ultrapure water, the sample was loaded. The column was washed with 4.5 ml H_2_O/MeOH (65:35; v/v) and retained compounds were eluted with 2.5 ml H_2_O/MeOH (20:80; v/v) into a 10 ml test tube and evaporated to dryness under nitrogen at 60°C using a nitrogen evaporator. The sample was finally reconstituted in 100 μL H_2_O/MeOH (80:20; v/v) in a vial and analyzed by means of UPLC-MS/MS.

Glucocorticoids were separated using a gradient elution of mobile phases A and B. Mobile phase A was a mixture of ultrapure water with 0.1% formic acid, while mobile phase B was a mixture of methanol with 0.1% formic acid. Initially, gradient elution started at 20% (v/v) of mobile phase B. Subsequently, mobile phase B was increased to 56% at 1.5 min, to 63% at 6.5 min, to 99.1% at 7.5 min after which it was kept at 99.1% to 8 min, and finally decreased to 20% at 9 min and kept in this way to 10 min. The flow rate was kept constant at 0.4 ml min^-1^, resulting in a 10 min running time. Samples were cooled at 7°C in the autosampler. The injection volume was set at 40 μL, while the column temperature was maintained at 30°C. Chromatographic analysis was performed on a mass spectrometer used in the multiple reaction monitoring (MRM) mode in order to achieve optimal sensitivity and selectivity. For cortisol as well cortisol-d_4_ two precursor fragment ion transitions were determined. Instrumental parameters were optimized by direct infusion of a 10 μg L^-1^ standard solution in methanol/0.1% formic acid at a flow rate of 10 μL min^-1^. The use of two fragment ion transitions allowed the determination of the ratio between both transitions, which was used together with the relative retention time for the identification and confirmation of the identity of each compound according the requirements of the Commission Decision No. 2002/657/EC. The mass spectrometer was used in positive electrospray ionization mode (ESI^+^). Both compounds were analyzed as their proton adducts [M+H]^+^. The MS detector settings were set at the following values: a source temperature of 120°C, a de-solvation temperature of 300°C at a gas flow of 800 L h^-1^, a cone gas flow of 50 L h^-1^, and a capillary voltage of 3 kV. Argon was used as collision gas at a pressure of 1.11 10^–2^ mbar. The optimized UPLC-MS/MS conditions with indication of retention time, precursor ion, fragment ions, cone voltage, and collision energy for both compounds are given in Table 1.

**Table 1:** Optimized UPLC-MS/MS conditions per compound.

Compound	Retention time	Precursor ion	Fragment ion—quantification and qualification trace	Cone voltage	Collision energy
	(min)	(m/z)	(m/z)	(V)	(eV)
Cortisol-d_4_	3.14	367.2	121.10*	35	20
			331.30		18
Cortisol	3.15	363.2	121.10*	35	20
			327.30		18

* = quantifier ion

Data analysis was performed using Quanlynx software from Waters; analysis results were reported as the value (μg kg^-1^) ± the expanded measurement uncertainty (μg kg^-1^) with a coverage factor (k) of 2 (95% confidentiality interval).

The UPLC-MS/MS method for cortisol in fish scales was validated according the requirements of the Commission Decision No. 2002/657/EC (S1 Protocol [52–59]).

### Gene expression analyses

To assess relative expressions of target genes, total RNA was isolated from the respective tissues with Trizol reagent (Invitrogen Life Technologies, Carlsbad, CA, USA) according to the manufacturer’s instructions. Tissues were homogenized in Trizol reagent using an MM300 bead laboratory mixer mill (Retsch GmbH, Haan, Germany) for 30 s at 20 Hz. Quality and quantity of RNA was assessed with a Nano-Drop ND-1000 spectrophotometer (NanoDrop Technologies, Wilmington, DE, USA); ethidium bromide-stained agarose gel separation was used to evaluate RNA integrity. All samples were then standardized to a RNA concentration of 500 ng μl^-1^ and subjected to treatment with DNAse I (Invitrogen), following the manufacturer’s instructions, prior to cDNA synthesis. Reverse transcription (RT) was performed in a final volume of 20 μl with first strand buffer, 5 mM DTT, 200 nM dNTPs, 300 ng random primers, 20 U RNaseOUT (Invitrogen) and 200 U SuperScript II reverse transcriptase (Invitrogen). No-template (NTC) and no-reverse transcriptase controls (NRC) were included for quality assessment. RT reactions were performed at 42°C for 50 min followed by 70°C for 15 min. Five μl of 5-times diluted cDNA from each RT reaction was used as template for real-time polymerase chain reaction (qPCR). The reaction mixture further contained SYBR Green PCR Master Mix (Applied Biosystems, Foster City, CA, USA) and 500 nM of each primer (primer sequences given in S1 Table). Real-time PCR was performed on a C1000 thermal cycler with CFX96 optical detection module (Bio-Rad, Hercules, CA, USA). Reaction conditions were 95°C for 10 min, 40 cycles of 95°C for 15 s and 60°C for 1 min. Target gene mean normalized expression (MNE) was determined using a normalization factor calculated by geNorm software [60], based on three selected housekeeping genes, *i*.*e*. elongation factor 1α (*elf1a*), 40S ribosomal protein S11 (*40s*) and beta-actin (*b-act*). PCR efficiencies were calculated from standard curves derived from serial dilutions (500–15.6 ng) of a RNA pool from randomly selected samples and were always in the range of 1.92–2.11.

### Scale morphometric analyses

Scale morphometric analyses [61] of regenerated scales were performed: (i) a decrease in total scale surface area indicates impaired growth; (ii) alterations of perimeter and (iii) a decrease in circularity indicates impaired growth and/or remodeling; while (iv) the number of ridges (radii) is indicative of regenerating scale growth rate.

### Statistical analyses

All parameters were modeled using a linear mixed model in SAS 9.4 (SAS Institute Inc., Cary, NC) with treatment, day of sampling and their interaction (where appropriate) as fixed effects. A random intercept for tank was introduced in the model to correct for clustering of fish in tanks. Dunnett’s test was performed to compare treatments with controls.

## Results and Discussion

### Plasma cortisol is a poor predictor of chronic stress exposure

During a 6-weeks trial, we compared undisturbed (CTR) and daily stressed (STRESS) carp. Dexamethasone (DEX) or cortisol (CORT) fed fish served as negative and positive controls for cortisol incorporation in the scale, respectively. In all treatments the feed ration provided was consumed within 5 minutes, all fish remained clinically healthy as indicated by the condition factor [46] and no morbidity nor mortality was observed. However, DEX fish showed a significantly lower condition factor (Table 2) in line with the expected effect of dexamethasone on the health status of the fish.

**Table 2:** Growth parameters analysed: D21 length (n = 48, F_(3,40)_ = 1.09, p = 0.3641), D21 weight (n = 48, F_(3,40)_ = 0.71, p = 0.5516), D21 condition factor (n = 48, F_(3,40)_ = 5.29, p = 0.0036), D42 length (n = 47, F_(3,39)_ = 1.30, p = 0.2884), D42 weight (n = 47, F_(3,39)_ = 0.90, p = 0.4500), and D42 condition factor (n = 47, F_(3,39)_ = 7.37, p = 0.0005).

		Average value			
Day	Parameter	CTR	DEX	CORT	STRESS
21	Length (cm)	24.83	25.58	24.33	23.75
21	Weight (g)	366.17	347.75	337.33	306.67
21	Condition factor	2.30	2.08**	2.33	2.28
42	Length (cm)	25.83	26.21	25.29	24.27
42	Weight (g)	407.75	382.50	387.25	328.64
42	Condition factor	2.27	2.08*	2.37	2.29

* = p<0.05,

** = p<0.01,

*** = p<0.001

Adequate water quality was maintained by daily (pH, temperature, dissolved oxygen) or weekly (nitrite, nitrate, ammonium) monitoring. A contribution of waterborne glucocorticoids, as a result of leakage from feed or anthropogenic contamination, to the effects observed, can be excluded. Analyses of cortisol, its precursors and metabolites by an in-house developed and validated UPLC-MS/MS method revealed that elevated cortisol concentrations in water returned to baseline within 2.5 h after feeding (data not shown).

**Fig 1:**
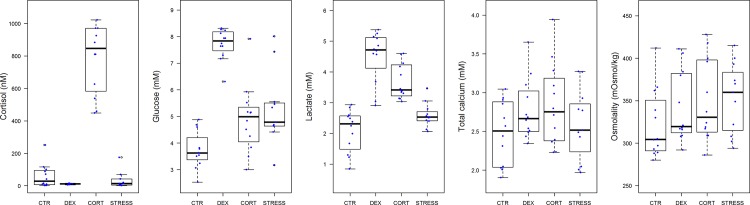
Plasma analyses of cortisol (nM) (n = 47, F_(3,39)_ = 12.80, p<0.0001), glucose (mM) (n = 47, F_(3,39)_ = 13.12, p<0.0001), lactate (mM) (n = 47, F_(3,39)_ = 16.01, p<0.0001), total calcium (mM) (n = 47, F_(3,39)_ = 1.59, p = 0.2068), and osmolality (mOsmol kg-1) (n = 47, F_(3,39)_ = 0.69, p = 0.5655) at day 42 of treatment. Fifty percent of the observations occurs between the lower and upper edges of the box (the first and third quartiles) and the whiskers extend to the most extreme observation which is no more than 1.5 times the interquartile range from the box; open circles represent values outside the range mentioned. For statistics, see S2 Table.

Results for five blood parameters commonly used as stress indicators confirm that DEX fish may be considered as negative, and CORT fish as positive controls, respectively (Fig 1). Plasma cortisol, the most commonly used parameter for stress, had elevated in the CORT group (CORT vs. CTR: *P*<0.0001), confirming the absorption of cortisol from feed into the blood; in STRESS fish cortisol levels did not differ from negative controls indicating the unsuitability of the latter for chronic stress assessment. The secondary stress parameter plasma glucose, reflecting predicted glucocorticoid actions (gluconeogenesis), had increased in DEX (DEX vs. CTR: *P*<0.0001), while CORT (CORT vs. CTR: *P* = 0.2004) and STRESS (STRESS vs. CTR: *P* = 0.0531) did not differ significantly, in line with the more potent action of DEX. Plasma lactate, an indicator of anaerobic metabolism and fatigue, had increased in DEX (DEX vs. CTR: *P*<0.0001) and CORT (CORT vs. CTR: *P* = 0.0004), but not significantly in STRESS (STRESS vs. CTR: *P* = 0.4224) in line with the downstream effects of DEX and CORT. Fish regulate calcium levels tightly (ionic, physiologically important calcium more strictly than total calcium) as calcium is pivotal to vision, muscle contraction, signal transduction, membrane permeability, etc. Even minor disruptions induce stress and disturbance of calcium balance. Plasma osmolality discloses disturbances in water and ionic balance, characteristic aspects of stress in fish which occur due to the intimate relationship between the body fluids in the gills and the aquatic environment. Plasma total calcium and osmolality were not affected indicating that the treatments did not exceed the resilience of the fish and did not evoke distress [13, 18–19]. We conclude that (i) CTR, DEX and CORT treatments elicited the intended glucocorticoid actions; (ii) in plasma of STRESS fish no signs of activation of HPI-axis were observed; (iii) plasma parameters are poor predictors for chronic stress as they reflect no more than a snap-shot of the stress response at a given moment.

**Fig 2:**
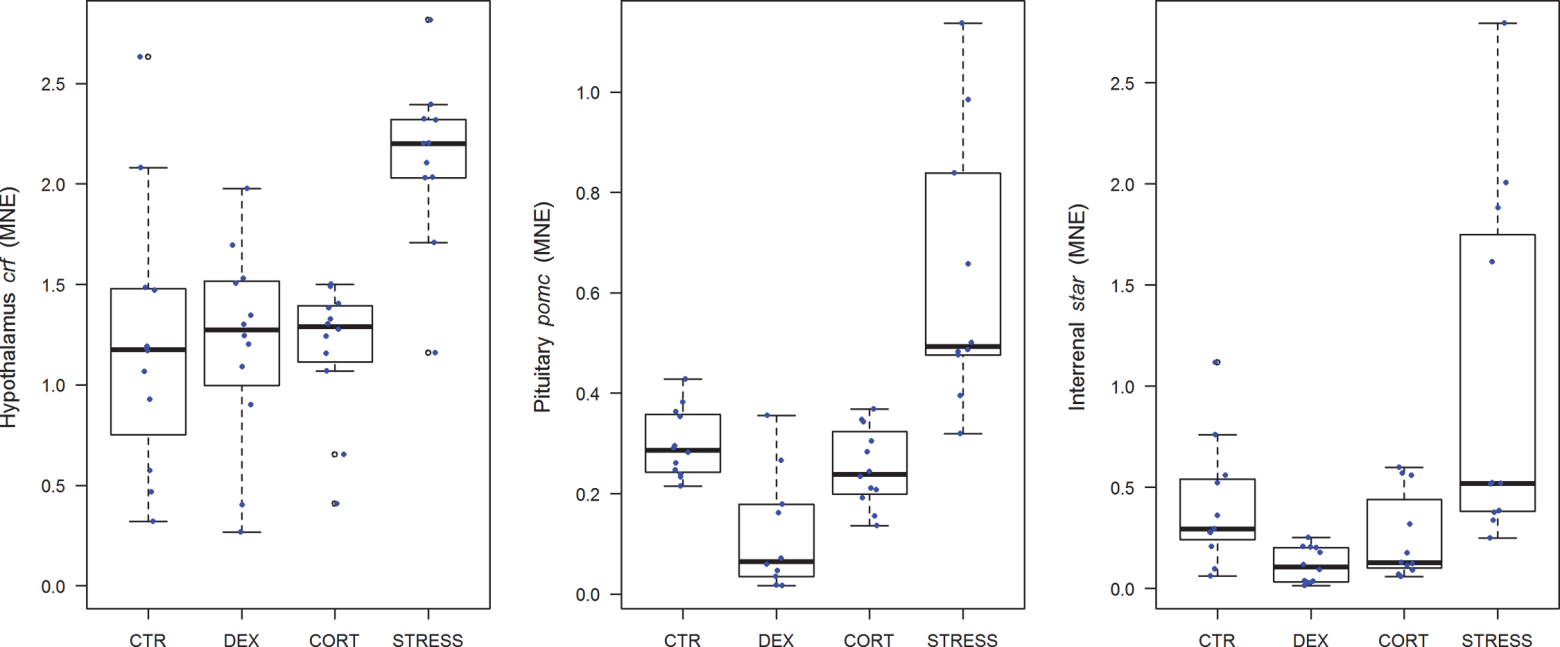
Analyses of hypothalamic (*crf*) (n = 47, F_(3,39)_ = 9.71, p<0.0001), pituitary pars distalis (*pomc)* (n = 44, F_(3,36)_ = 20.83, p<0.0001), and interrenal (*star*) (n = 45, F_(3,37)_ = 7.75, p = 0.0004) genes (MNE) at day 42 of treatment. Error bars in scatter/boxplots are defined as in Fig 1. For statistics, see S2 Table.

From the above it follows that plasma cortisol values in chronically stressed fish do not provide an adequate read-out for stress experienced. However, Fig 2 shows that the endocrine stress axis is most certainly activated. CRF (corticotropin-releasing factor) stimulates corticotrope cells in anterior pituitary, or pars distalis, to secrete POMC (pro-opiomelanocortin), a prohormone that gives rise to multiple peptide hormones among which is ACTH (adrenocorticotropic hormone), the peptide hormone that stimulates the secretion of cortisol from the interrenal cells. Steroidogenic acute regulatory (StAR) protein is a transport protein that regulates cholesterol transfer within the mitochondria, which is together with p450scc the rate-limiting step in the production of steroid hormones. The expression of hypothalamic, pituitary pars distalis, and interrenal key genes *crf* (STRESS vs. CTR: *P* = 0.0002), *pomc* (STRESS vs. CTR: *P*<0.0001), and *star* (STRESS vs. CTR: *P* = 0.0140), respectively, were significantly up-regulated indeed. Negative glucocorticoid feedback is indicated by significantly decreased *pomc* expression (DEX vs. CTR: *P* = 0.0236).

**Fig 3:**
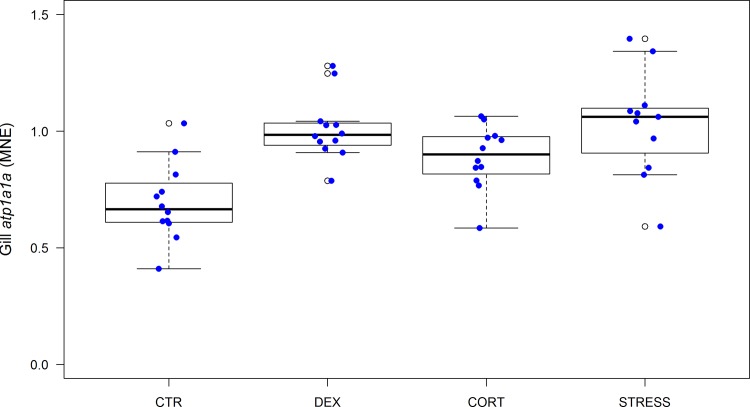
Gene expression of *atp1a1a* in gills (MNE) (n = 47, F_(3,39)_ = 9.76, p<0.0001) after 42 days of treatment. Error bars in scatter/boxplots are defined as in Fig 1. For statistics, see S2 Table.

Gills play a crucial role in hydromineral homeostasis of a fish. In freshwater fish, the function of chloride cells in the branchial epithelium is uptake of ions (Na, Cl, Ca) from the surrounding water. The most important and extensively studied enzyme in the chloride cell is sodium/potassium-activated adenosine triphosphatase (Na^+^/K^+^-ATPase), the enzymatic expression of the sodium pump. A second line of evidence for chronic stress, not visible at the level of plasma cortisol, is the up-regulation of the gene encoding the subunit α1 of the branchial Na^+^/K^+^-ATPase [62] (Fig 3). In DEX (DEX vs. CTR: *P* = 0.0001), CORT (CORT vs. CTR: *P* = 0.0215) and STRESS (STRESS vs. CTR: *P*<0.0001) fish, significantly enhanced expression levels were found, indicative of corticoid action.

In summary, results indicate that fish of the STRESS group were chronically stressed.

### Cortisol in ontogenetic scales of fish as biomarker for chronic stress

Five (day 21) to six (day 42) scales (total average weight of 0.1285 g and 0.1308 g, respectively) were analysed for cortisol with a validated UPLC-MS/MS method.

**Fig 4:**
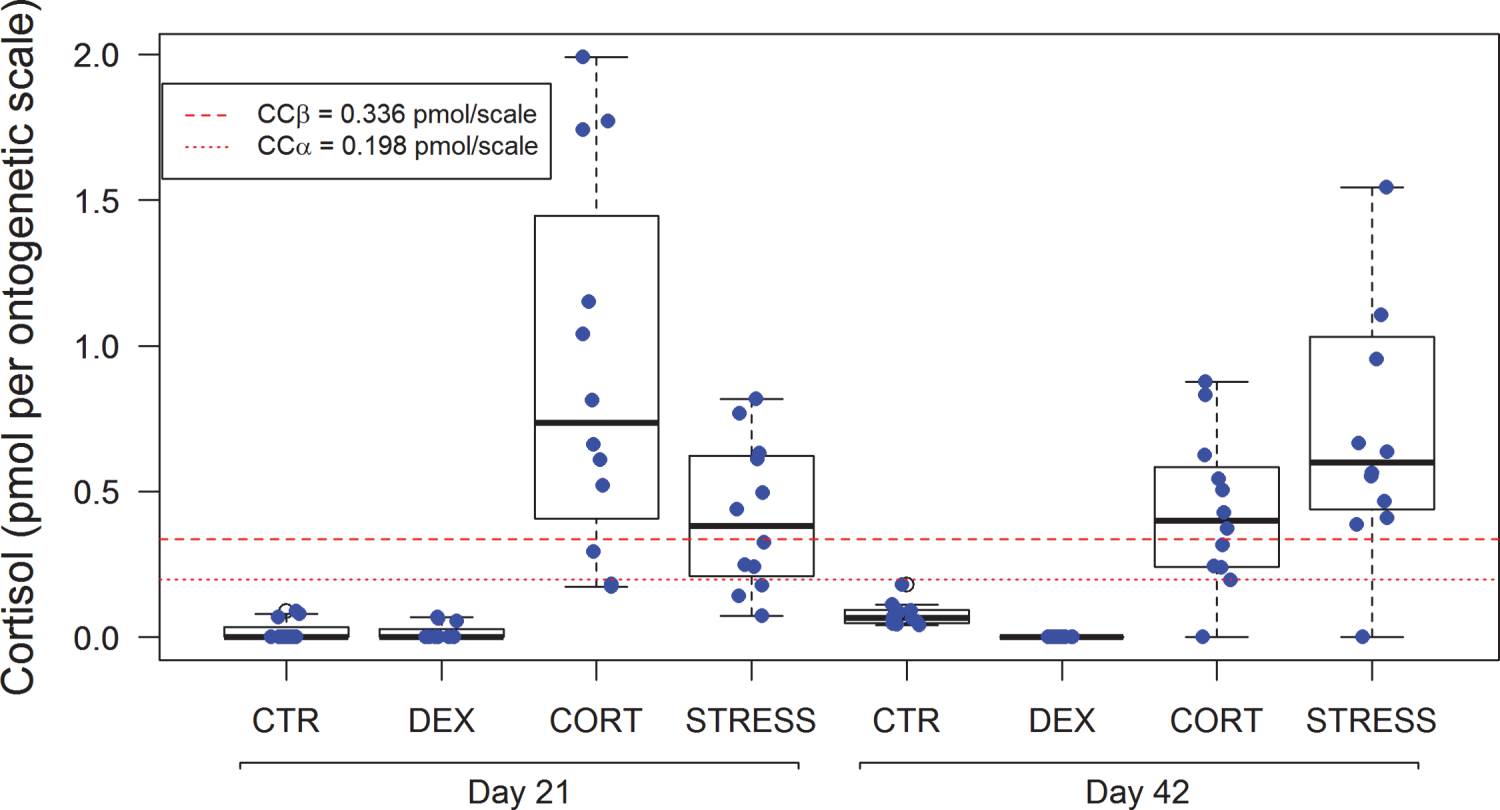
Cortisol (pmol per scale) in ontogenetic scales after 21 and 42 days of treatment (CCα = decision limit and CCβ = detection capability were calculated using an average weight of 0.026 g per scale). One value (2.8 pmol per scale) of STRESS day 42 was omitted in the scatter/boxplot for reasons of presentation. Error bars in scatter/boxplots are defined as in Fig 1. For statistics, see S2 Table.

All CTR and DEX fish showed scale cortisol values below the detection capability (CCβ), while most STRESS and CORT fish showed scale cortisol values above CCβ at both days (Fig 4).

Upon comparing treatments, at day 21 a significant accumulation was found for CORT fish (CORT vs. CTR: *P*<0.0001); at day 42 a significant accumulation was found for STRESS fish (STRESS vs. CTR: *P* = 0.0001). When comparing within treatments from 21 to 42 days, a significant increase in scale cortisol was found in STRESS (*P* = 0.0031) and CORT (*P* = 0.0026) fish, respectively. These findings are in line with the predicted incorporation and accumulation of cortisol in scales over time and validate the scale cortisol content as a biomarker for chronic stress. In line with the predicted effect of blocking endogenous cortisol production by DEX, we found no cortisol incorporation in scales of DEX treated fish. On the other hand, feeding cortisol and physically stressing the fish enhanced scale cortisol levels.

In summary, the results for cortisol in ontogenetic scales confirmed our initial hypothesis that cortisol in scales reflect the stress level experienced by fish over time.

### Cortisol also accumulates in regenerating scales

**Fig 5:**
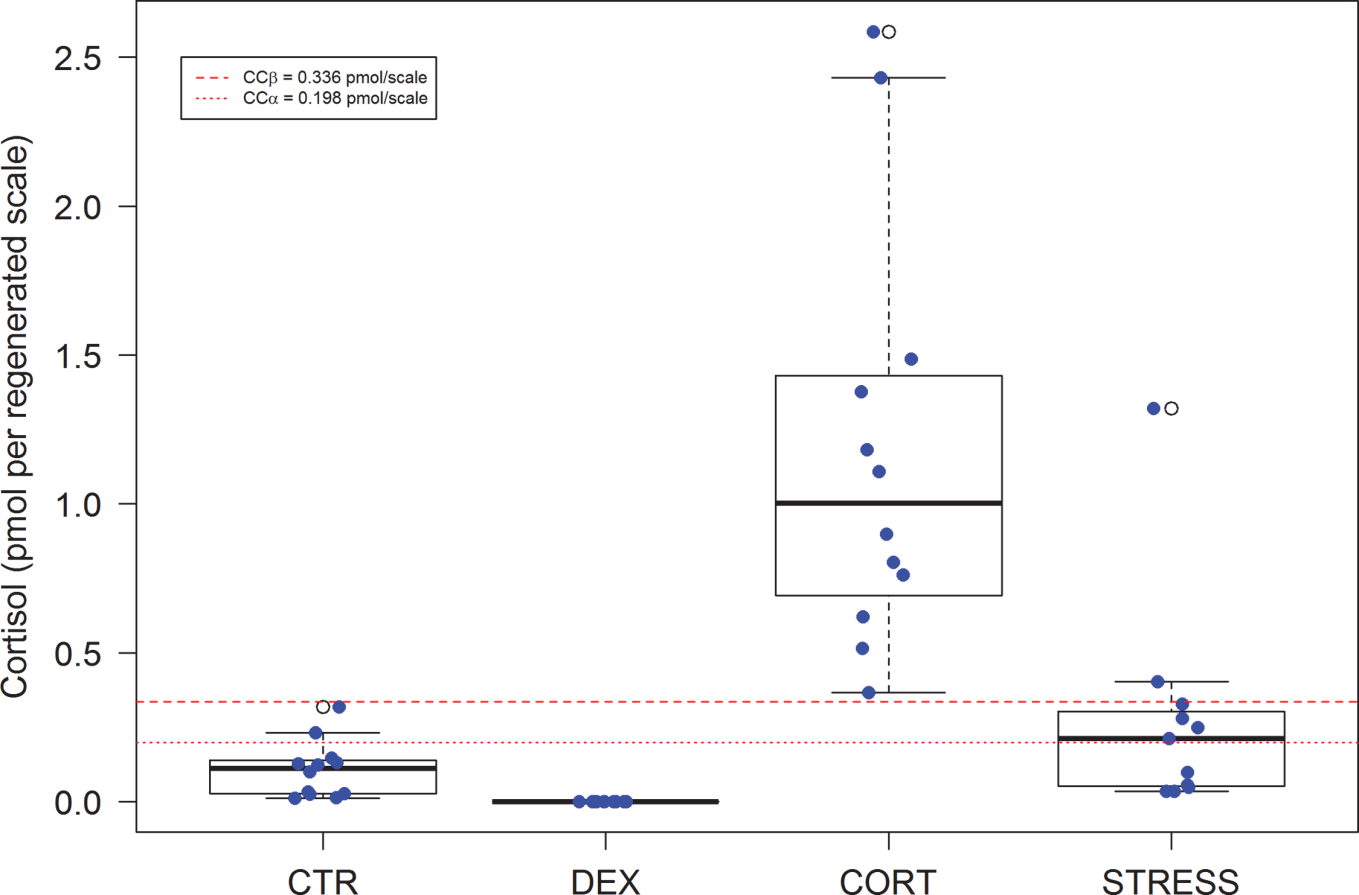
Cortisol (pmol per scale) in regenerated scales of 21 days (n = 48, F_(3,40)_ = 10.16, p<0.0001) (CCα = decision limit and CCβ = detection capability were calculated using an average weight of 0.026 g per scale). Error bars in scatter/boxplots are defined as in Fig 1. For statistics, see S2 Table.

Upon removal, a scale will regenerate within days (41). Cortisol concentrations and scale morphometric analysis were performed in 5 regenerated scales (total average weight of 0.0211 g) per fish 21 days after removal of the ontogenetic scale (Fig 5).

**Fig 6:**
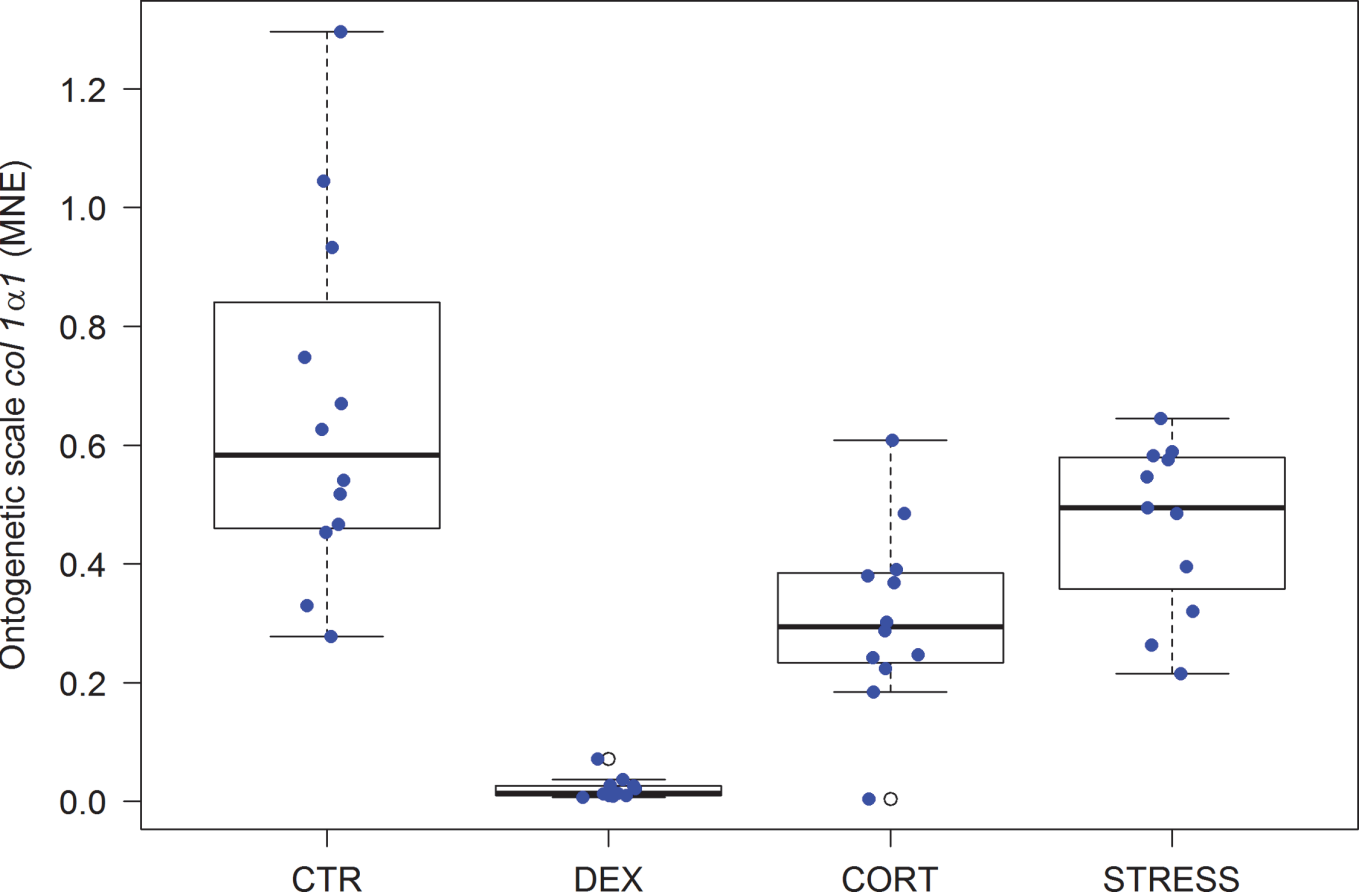
Gene expression of *col 1a1* in ontogenetic scale (MNE) (n = 47, F_(3,39)_ = 23.70, p<0.0001) after 42 days of treatment. Error bars in scatter/boxplots are defined as in Fig 1. For statistics, see S2 Table.

The MNE of *col1a1*, the gene encoding collagen type-1, the most abundant scale matrix protein, was found to be inhibited in ontogenetic scales (CORT vs. CTR: *P* = 0.0002, see Fig 6) and correlates with high plasma cortisol level (CORT vs. CTR: *P*<0.0001, see Fig 1), in line with the well-known inhibitory effects of glucocorticoids on bone formation [63].

In regenerating scales, the scleroblasts (scale-forming osteoblast-like cells [41]) appear insensitive to glucocorticoid feedback as shown by similar *col1a1* expression in all treatments. For statistics, see S2 Table.

Morphologically, regenerated scales of CTR, STRESS, and CORT were found to be similar, while the area, perimeter and number of ridges of scales of DEX fish showed significantly lower values indicative of disturbed and retarded regeneration (Table 3).

**Table 3:** Regenerated scale morphometric analyses: area (n = 47, F_(3,39)_ = 13.22, p<0.0001), perimeter (n = 47, F_(3,39)_ = 5.65, p = 0.0026), circularity (n = 47, F_(3,39)_ = 0.36, p = 0.7812), and ridges (n = 47, F_(3,39)_ = 3.03, p = 0.0406).

		Average value			
Day	Parameter	CTR	DEX	CORT	STRESS
42	Area (A) per regenerated scale (cm^2^)	1.05	0.59***	0.98	0.85
42	Perimeter (P) per regenerated scale (cm)	4.39	3.41**	4.31	4.04
42	Circularity (4πA/P^2^) regenerated scale	0.68	0.62	0.66	0.66
42	Ridges per regenerated scale (n)	14.25	12.58*	13.08	13.09

* = p<0.05,

** = p<0.01,

*** = p<0.001

In summary, the high cortisol plasma level in CORT fish at the moment of sampling of regenerating and ontogenetic scales at day 42 correlates with a high scale cortisol content, confirming our hypothesis that free cortisol incorporates in scales. Furthermore, regenerated scale cortisol content in STRESS fish appeared to be as yet not significantly higher than of CTR but lower than CORT, indicating that the maximal sequestering of cortisol in the matrix had not been reached. A significant increase in scale cortisol in CORT fish (CORT vs. CTR: *P*<0.0001) was observed. This finding is in line with the predicted incorporation and accumulation of cortisol in scales over time with higher plasma cortisol levels available, hereby reconfirming our findings on ontogenetic scales. The results for regenerated scales corroborate the results for ontogenetic scales and thus our initial hypothesis that cortisol in scales reflects the stress level experienced by fish in time.

## Conclusion

The pertinent literature lacks data on cortisol in a matrix that captures systemic cortisol exposure over longer periods of time suitable for chronic stress evaluation in fish. The assay of cortisol in plasma as well as in alternative matrices such as feces, mucus and water have all restrictions regarding its suitability for assessment of chronic stress.

We showed that the scale cortisol content is highly suitable for quantification of chronic stress in common carp, this notion makes scale cortisol an innovative and beyond state-of-the art biomarker with high potential impact in science and industries related to fish (*e*.*g*. physiology, toxicology, immunology, behavioral studies, *etc*.). The findings presented for carp seem applicable for all fish with elasmoid scales as we obtained comparable data for highly relevant species for aquaculture such as sea bass (*Dicentrarchus labrax*) and Mozambique tilapia as well as for fish commonly used in experimental studies such as zebrafish (*Danio rerio*). In a broader sense placoid and ganoid scales are predicted to be suitable as well expanding the use of this biomarker to other actinopterygian and even chondrichtyan species. Finally, this new tool for chronic stress monitoring has a huge valorization potential to be adopted in a broad range of governmental, academic and industrial settings such as decision making on animal friendly aquaculture (*e*.*g*. animal-based optimization of Recirculating Aquaculture Systems and improvement of product quality), monitoring of wild fish stocks (*e*.*g*. ecology based population dynamics), the welfare of fish in public aquaria and in animal trials. The development of a financially, logistically and widely applicable feasible assay for on-site analysis of cortisol in scales of fish has been initiated.

## Supporting Information

S1 Protocol Validation of scale cortisol. (DOCX)

S1 Table Primer sequences used for qPCR. (DOCX)

S2 Table Overview of parameters analysed. * = p<0.05, ** = p<0.01, *** = p<0.001.
(DOCX)
