# Supplementary material for: Scales Tell a Story on the Stress History of Fish
Source: PLoS One. 2015 Apr 29;10(4):e0123411. doi: 10.1371/journal.pone.0123411 (PMC4414496; doi:10.1371/journal.pone.0123411)
Supplement: S1 Protocol — (DOCX) [file pone.0123411.s001.docx]

**Protocol S1. Validation of scale cortisol.**

Validation samples were made by pooling of scales from 51 fish. No certified reference material, inter-laboratory comparison tests or any other validated methods for the above mentioned compound/matrix combinations exist, and therefore validation was done using standard addition to validation samples. Five concentration levels, ranging from 1 to 50 µg kg^-1^, were tested in five-fold and this was repeated on four different days within a period of one month under intra-reproducibility conditions, *i.e.* by two persons using different solutions and one UPLC-MS/MS system.

All validation experiments were carried out by authorized personnel in a controlled environment with calibrated equipment and controlled solutions according to the requirements of the standard EN ISO/IEC 17025 [50]. Analysis was done using standardized sequences consisting of different calibration standards, blanks, negative and positive controls (all in diluent). Results for every compound were evaluated by assessing the (relative) retention time and relative ion intensities of the compound and fragments.

All validation parameters were determined and evaluated according the requirements of the Commission Decision No. 2002/657/EC [51].

The working range for validation was set from 1 µg kg^-1^ to 50 µg kg^-1^, based on pre-validation analyses of scales from highly relevant species for aquaculture such as common carp, sea bass (*Dicentrarchus labrax*), Mozambique tilapia (*Oreochromis mossambicus*), Atlantic salmon (*Salmo salar*) and pike perch *(Stizostedion lucioperca*) as well as for fish commonly used in experimental studies such as zebrafish (*Danio rerio*).

Since in future research matrix-matched calibration curves are not practically feasible, calibration curves were made in diluent. The linearity of the response of cortisol in scales was determined by comparison of the experimental and theoretical curve and tested using four-fold calibration curves in diluent consisting out of five calibration points, ranging from 5 µg kg^-1^ to 100 µg kg^-1^, analyzed under intra-laboratory reproducibility conditions. When assessing the linearity in this range for cortisol a coefficient of determination (R^2^) value of 0.999 was found. Furthermore the calculated model indicated a normal distribution.

The apparent recovery as well as precision (repeatability and intra-laboratory reproducibility) for all compounds were determined under intra-reproducibility conditions resulting in 20 analyses for every concentration level and a total of 100 analyses per compound. Due to the low concentration levels (in particular 1 µg kg^-1^), a Dixon’s outlier test [52] as well as a Grubbs’ [53] test were performed to detect possible outliers. The apparent recovery for cortisol in fish scales below, at and above CCβ level ranged from 88.14 % to 98.85 %, all within the performance criterion according to the requirements of the Commission Decision No. 2002/657/EC. Despite the fact that the homogenization of the pooled validation sample was not perfect (*i.e.* it was not a homogenous powder, but rather a pool of very fine scale fragments), the repeatability for cortisol in fish scales ranged from 12.36 % to 17.15 %. The intra-laboratory reproducibility ranged from 14.63 % to 20.02 %. The expanded measurement uncertainty was determined by linear summation as well as by quadratic summation or Nordtest method [54-59] as there is no consensus in the literature on a preferred method. The results for trueness (apparent recovery AR, percent), coefficient of variation values (CV, percent) for precision (repeatability CVr and intra-laboratory reproducibility CV_R_) and expanded measurement uncertainty (U, percent) for cortisol in fish scales are presented in following table.

| Level | AR | CV_r_ | CV_R_ | U^1^(k=2) | U^1^(k=3) | U^2^(k=2) |
| --- | --- | --- | --- | --- | --- | --- |
| (µg kg^-1)^ | (%) | (%) | (%) | (%) | (%) | (%) |
| 1 | 98.85 | 17.15 | 20.02 | 41.19 | 61.21 | 66.16 |
| 5 | 88.14 | 12.36 | 15.01 | 41.88 | 56.89 | 58.08 |
| 10 | 98.20 | 16.21 | 18.05 | 37.90 | 55.95 | 61.44 |
| 25 | 90.97 | 13.81 | 14.85 | 38.73 | 53.58 | 56.08 |
| 50 | 91.16 | 13.47 | 14.63 | 38.10 | 52.73 | 55.57 |

^1^ Calculation of the expanded measurement uncertainty using linear summation with a coverage factor (k) of 2 (95 % confidentiality interval) respectively of 3 (99 % confidentiality interval).

^2^ Calculation of the expanded measurement uncertainty using quadratic summation (Nordtest method) with a coverage factor (k) of 2 (95 % confidentiality interval).

The decision limit (CCα) was calculated as the intercept of the calibration curve plus 2.33 times the standard deviation on the intra-laboratory reproducibility (α = 1 %), while the detection capability (CCβ) was calculated as the concentration of CCα plus 1.64 times the standard deviation on the intra-laboratory reproducibility (β = 5 %). For cortisol CCα was 2.74 µg kg^-1^ and CCβ was 4.64 µg kg^-1^. The accepted CCβ concentration was adopted as the lowest standard on the working curve for routine analysis.

The sensitivity of the method was good for cortisol in fish scales in the predicted working range and determined by a dilution experiment on blank matrix samples.

The selectivity of the method was tested by analyzing blank and spiked (using compounds with similar physical and chemical properties; *i.e.* precursor and metabolites of cortisol) samples. The selectivity was proven as in spiked samples, cortisol appeared at predicted retention time with their two ion transitions. The ion ratios were compared with the standard injection and were found acceptable according to the requirements of the Commission Decision No. 2002/657/EC. Furthermore no interfering peaks were observed.

The robustness of the method and stability of the compounds in diluent as well as in matrix were monitored using trendcharts during method development and subsequent validation.
