# Supplementary material for: Scales Tell a Story on the Stress History of Fish
Source: PLoS One. 2015 Apr 29;10(4):e0123411. doi: 10.1371/journal.pone.0123411 (PMC4414496; doi:10.1371/journal.pone.0123411)
Supplement: S1 Table — (DOCX) [file pone.0123411.s002.docx]

**Table S1. Primer sequences used for qPCR.**

| **Gene** | **Accession no.** | **Sequence (5’ → 3’)** |
| --- | --- | --- |
| *elf1a* | AF485331 | fw: CACGTCGACTCCGGAAAGTC |
|  |  | rv: CGATTCCACCGCATTTGTAGA |
| *40s* | AB012087 | fw: CCGTGGGTGACATCGTTACA |
|  |  | rv: TCAGGACATTGAACCTCACTGTCT |
| *b-act* | M24113 | fw: CAACAGGGAAAAGATGACACAGATC |
|  |  | rv: GGGACAGCACAGCCTGGAT |
| *crf* | AJ317955 | fw: CATCCGGCTCGGTAACAGAA |
|  |  | rv: CCAACAGACGCTGCGTTAACT |
| *pomc* | Y14618 | fw: TTGGCTCTGGCTGTTCTGTGT |
|  |  | rv: TCATCTGTCAGATCAGACCTGCATA |
| *star* | EU519825 | fw: CAAACCCAGGTGGATTTCGC |
|  |  | rv: TCGCCCTTGTTATGGCATCT |
| *col1a1* | AB685219 | fw: TGCAACCAGGATGCCATCAA |
|  |  | rv: GGACTCAGTTGGGTAGACGC |
| *atp1a1a* | AF286372 | fw: CAAGGTGGACAACTCCTCCC |
|  |  | rv: CTTCAAGGCCAGAAGCGAGA |
