# Supplementary material for: Scales Tell a Story on the Stress History of Fish
Source: PLoS One. 2015 Apr 29;10(4):e0123411. doi: 10.1371/journal.pone.0123411 (PMC4414496; doi:10.1371/journal.pone.0123411)
Supplement: S2 Table — * = p<0.05, ** = p<0.01, *** = p<0.001. (DOCX) [file pone.0123411.s003.docx]

**Table S2. Overview of parameters analysed.**

|  | | Average value | | | |
| --- | --- | --- | --- | --- | --- |
| Day | Parameter | CTR | DEX | CORT | STRESS |
| Plasma markers | | | | | |
| 42 | Plasma cortisol (nM) | 59.41 | 11.45 | 863.95*** | 35.40 |
| 42 | Plasma glucose (mM) | 3.74 | 7.72*** | 4.90 | 5.28 |
| 42 | Plasma lactate (mM) | 2.09 | 4.49*** | 3.69*** | 2.60 |
| 42 | Plasma total calcium (mM) | 2.48 | 2.78 | 2.84 | 2.59 |
| 42 | Plasma osmolality (mOsmol kg^-1^) | 322.08 | 342.50 | 350.25 | 353.36 |
| Key genes | | | | | |
| 42 | Hypothalamus *crf* (MNE) | 1.21 | 1.21 | 1.18 | 2.12*** |
| 42 | Pituitary *pomc* (MNE) | 0.30 | 0.12* | 0.25 | 0.63*** |
| 42 | Head Kidney *star* (MNE) | 0.41 | 0.12 | 0.25 | 1.02* |
| 42 | Gill *atp1a1a* (MNE) | 0.70 | 1.01*** | 0.89* | 1.03*** |
| Ontogenetic scale | | | | | |
| 21 | Cortisol (pmol per ontogenetic scale) | 0.020 | 0.016 | 0.912*** | 0.414* |
| 42 | Cortisol (pmol per ontogenetic scale) | 0.079 | 0.000 | 0.431* | 0.918*** |
| 42 | Ontogenetic scale *col1a1* (MNE) | 0.66 | 0.02*** | 0.31*** | 0.46 |
| Regenerated scale | | | | | |
| 42 | Cortisol (pmol per regenerated scale) | 0.11 | 0.00 | 1.18*** | 0.28 |
| 42 | Regenerated scale *col1a1* (MNE) | 1.29 | 1.12 | 1.08 | 1.37 |

*=p<0.05, **=p<0.01, ***=p<0.001
